# Melatonin blunted the angiogenic activity in 3D colon cancer tumoroids by the reduction of endocan

**DOI:** 10.1186/s12935-023-02951-5

**Published:** 2023-06-19

**Authors:** Maryam Taghavi Narmi, Hanieh Mohajjel Shoja, Sanya Haiaty, Mahdi Mahdipour, Reza Rahbarghazi

**Affiliations:** 1 Department of Plant, Cell and Molecular Biology, Faculty of Natural Sciences, University of Tabriz, Tabriz, 51666-16471 Iran; 2 Infectious and Tropical Diseases Research Center, Tabriz University of Medical Sciences, Tabriz, Iran; 3 Stem Cell Research Center, Tabriz University of Medical Sciences, Tabriz, Iran; 4 Department of Applied Cell Sciences, Faculty of Advanced Medical Sciences, Tabriz University of Medical Sciences, Tabriz, Iran

**Keywords:** 3D Colon cancer tumoroids, Multiple cells, Melatonin, Angiogenesis, Endocan

## Abstract

**Background:**

Complexity and heterogeneity of the tumor niche are closely associated with the failure of therapeutic protocols. Unfortunately, most data have been obtained from conventional 2D culture systems which are not completely comparable to in vivo microenvironments. Reconstructed 3D cultures composed of multiple cells are valid cell-based tumor models to recapitulate in vivo-like interaction between the cancer cells and stromal cells and the oncostatic properties of therapeutics. Here, we aimed to assess the tumoricidal properties of melatonin on close-to-real colon cancer tumoroids in in vitro conditions.

**Methods:**

Using the hanging drop method, colon cancer tumoroids composed of three cell lines, including adenocarcinoma HT-29 cells, fibroblasts (HFFF2), and endothelial cells (HUVECs) at a ratio of 2: 1: 1, respectively were developed using 2.5% methylcellulose. Tumoroids were exposed to different concentrations of melatonin, from 0.005 to 0.8 mM and 4 to 10 mM, for 48 h. The survival rate was measured by MTT and LDH leakage assays. Protein levels of endocan and VEGF were assessed using western blotting. Using histological examination (H & E) staining, the integrity of cells within the tumoroid parenchyma was monitored.

**Results:**

Despite the reduction of viability rate in lower doses, the structure of tumoroids remained unchanged. In contrast, treatment of tumoroids with higher doses of melatonin, 4 and 10 mM, led to disaggregation of cells and reduction of tumoroid diameter compared to the non-treated control tumoroids (p < 0.05). By increasing melatonin concentration from 4 to 10 mM, the number of necrotic cells increased. Data showed the significant suppression of endocan in melatonin-treated tumoroids related to the non-treated controls (p < 0.05). According to our data, melatonin in higher doses did not alter protein levels of VEGF (p > 0.05).

**Conclusions:**

Melatonin can exert its tumoricidal properties on colon cancer tumoroids via the reduction of tumor cell viability and inhibition of the specific pro-angiogenesis factor.

## Introduction

Colorectal cancer (CRC) is currently one of the most common devasting changes in the gastrointestinal tract, leading to high-rate human mortality in the clinical setting [1]. It is believed that the metastasis of cancer cells to remote sites is a big challenge in patients subjected to chemotherapy protocols [2]. Because of prominent vascularization into tumor parenchyma, the possibility of tumor cell metastasis is increased to the other sites [3]. Therefore, therapeutic modalities and antitumor regimes should focus on the control of blood nourishment and vascularization (angiogenesis) into the CRC niche in the early stages to inhibit tumor mass expansion and metastasis to other organs [4, 5]. The phenomenon of angiogenesis is a fundamental and biological procedure that occurs during both pathological and physiological conditions [6]. To be specific, angiogenesis is the development of nascent vessels from pre-existing networks [7]. It is believed that the balance between pro- and anti-angiogenic factors can control vascularization outcomes [8, 9]. Of several pro-angiogenesis factors, endocan, also known as specific endothelial molecule (ESM-1), is sulfate proteoglycan and is released by both cancer cells and endothelial lineage in response to the hypoxic condition [10, 11]. This factor can provoke other angiogenesis-related factors after production and secretion into the extracellular matrix [12, 13]. The previous data have supported the fact that the up-regulation of endocan is associated with tumor cell metastasis and poor prognosis in cancer patients [14]. Commensurate with these comments, the control and regulation of endocan is an appropriate strategy for the control of vascularization rate within the cancerous parenchyma [15].

Melatonin is a pleiotropic hormone secreted by the pineal gland and other tissues such as the skin, liver, etc [16]. Owing to its chemical structure, N-acetyl-5-methoxy-tryptamine, melatonin possesses diverse biological activities in different tissues [17]. For instance, both the angiogenesis and anti-angiogenesis capacity of melatonin has been proven in physiological and pathological conditions [18]. Of note, the possible anti-angiogenesis role of melatonin has been indicated on tumor niche via the suppression of pro-angiogenesis factors such as VEGF, bFGF, etc. in in vivo conditions and 2D conventional culture systems [19].

Unfortunately, findings obtained in laboratory settings could not be efficiently translated into human medicine. One reason would be that most previously established cancer models are not eligible to completely recapitulate the mutual interaction between the cancer cells with stromal cells and in vivo-like conditions [20]. During the past years, the advent of organoid technology (tumoroids), a promising alternative culture model to the conventional 2D system, has led to significant progress in understanding complex cancer cell biology [21–23]. Upon embedment into the supporting matrix, cells within the tumoroids can in part, but not completely, mimic the in vivo-like conditions in which architecture and cellular function are relatively similar to the primary sites. These features result in the acquisition of valuable data which are comparable to the human body [5, 24].

To the best of our knowledge, the direct impact of melatonin has not been indicated on endocan levels in 3D tumor organoids [25]. Here, we aimed to investigate the possible effects of melatonin on 3D CRC tumoroid angiogenesis capacity via monitoring the levels of endocan in in vitro conditions. To this end, three human cell types including, CRC adenocarcinoma HT29 cells, human fetal foreskin HFFF2 fibroblasts, and human umbilical vein endothelial cells (HUVECs) were used for the development of in vivo-like CRC tumoroids and exposed to different concentrations of melatonin. Indeed, HT-29 cells are the main cancer cells that mimic the anaplastic phenotype. To support, the vascularization and extracellular matrix integrity, HUVECs and HFFF2 fibroblasts were also included in developed tumoroids [26–28]. It is suggested that the result of this study can help us to understand the anti-tumor activity of melatonin in 3D tumoroids via the inhibition of certain angiogenesis factors like endocan and VEGF.

## Materials and methods

### Ethical issues

All phases of this study were approved by the Local Ethics Committee of Tabriz University of Medical Sciences (IR.TBZMED.VCR.REC.1400.445).

### Cell culture

In this study, CRC tumoroids were developed using three different human cell lines including HUVECs, HT-29, and HFFF2 cells. Cells were purchased from Iranian National Cell Bank (Tehran, Iran) and cultured in RPMI-1640 culture medium (Cat no; 21875-034; Gibco) with 10% fetal bovine serum (FBS; Cat no: 26140-079; Gibco) and 1% Pen-Strep (Cat no; 10378-016; Gibco). Cells were cultured at the recommended standard condition at 37˚C with 5% CO_2_ and 95% relative humidity. Cells were sub-cultured upon reaching 70–80% confluence using 0.25% Trypsin-EDTA solution (Cat no; R001100; Gibco). In this study, cells at passages from three to six were used for subsequent analyses.

### Development of 3D CRC tumoroids

CRC tumoroids were generated using the hanging drop method as previously described with some modifications [29]. In this study, the total number of cells in each cluster was adjusted to 1 × 10^3^. HT-29, HFFF2 cells, and HUVECs were used at a ratio of 2: 1: 1, respectively. In short, the mixture of cells was resuspended in a 25 µl culture medium containing 1% FBS and 2.5% methylcellulose (Cat no: MO512; Sigma-Aldrich) and placed in the inner surface of a 10 cm culture dish lids (SPL). After that, the lids were carefully inversed and put into plates [29]. About 5–6 ml phosphate buffered saline (PBS) was poured onto the culture plates to prevent the drying of droplets until the analyses. Clusters were maintained for 72 h until stiff tumoroids were generated. The tumoroids were transferred using sterile yellow tips into the culture plates for different analyses.

### Melatonin treatment and survival assay

The oncostatic effects of melatonin were assessed on the CRC tumoroids in vitro using lactate dehydrogenase (LDH) Cytotoxicity Assay [30]. For this purpose, a single tumoroid was placed in a 200 µl culture medium with 1% FBS and gently transferred onto each well of 96-well culture plates. After 24 h, cells were exposed to different doses of melatonin ranging from 0.005 to 0.8 mM and 4, 6, 8, and 10 mM for 48 h. In this study, melatonin dissolved in dimethyl sulfoxide (DMSO), and the final concentration of solvent was below 1%. After the completion of incubation time, supernatants were collected and levels of LDH were determined using LDH Cytotoxicity Assay kits (Lot no: 99,003; Pars Azmun Co. Ltd, Iran). Supernatants were centrifuged at 300 g to eliminate the debris. Using recommended reagents and incubation time, the optical density was read at a wavelength of 340 nm.

### Tumoroids diameter and integrity

To monitor the integrity of CRC tumoroids, the average diameter of tumoroids was measured in each group using ImageJ software (NIH, Ver. 1.4) [31]. The values were compared to the non-treated control tumoroids.

### Hematoxylin-eosin staining

In this regard, tumoroids were embedded in a 1% agar solution. The procedure was continued by the incubation of embedded tumoroids in a 10% buffered formalin solution for 48 h. Paraffin-embedded blocks were cut into 5 μm thick sections. Slides were stained using Hematoxylin-Eosin (H & E) solution as previously described [32]. The structure and integrity of tumoroids parenchyma were monitored using Olympus microscopy.

### Monitoring the levels of endocan using western blotting

To monitor the angiogenesis status, protein levels of endocan and VEGF were measured in melatonin-treated tumoroids using western blotting. Tumoroids were gently washed with PBS, and centrifuged at 1500 rpm for 5 min. After discarding the supernatants, tumoroids were incubated with RIPA lysis buffer for 20 min. Samples were centrifuged at 14,000 rpm for 20 min and supernatants were collected and used for analyses. Samples (about 10 µg per group) were electrophoresed using 10% SDS-PAGE followed by transferring onto the PVDF membrane. After blocking with 1% bovine serum albumin (BSA; Sigma-Aldrich), membranes were incubated with anti-human endocan (Cat no: sc-515,304; Santa Cruz Biotech Inc., USA) and –VEGF (Cat no: sc-7269; Santa Cruz Biotech Inc., USA) antibodies for 1 h at RT. The membranes were washed three times with PBST (each in 10 min) and incubated with HRP-conjugated secondary antibodies at RT for 1 h. Membranes were again washed with PBS (3 × 10 min) and immunoreactive bands were visualized using an ECL solution and X-ray films. The density of each band was calculated using ImageJ software (NIH, ver.1.4) in comparison with the housekeeping protein β-actin.

### Statistical analysis

Data are presented as mean ± SD. Using One-way ANOVA with the Tukey post hoc test, the differences between groups were compared. p < 0.05 was considered statistically significant. All experiments were done in triplicate otherwise mentioned.

## Results

### Tumoroids morphology and survival rate

**Fig. 1:**
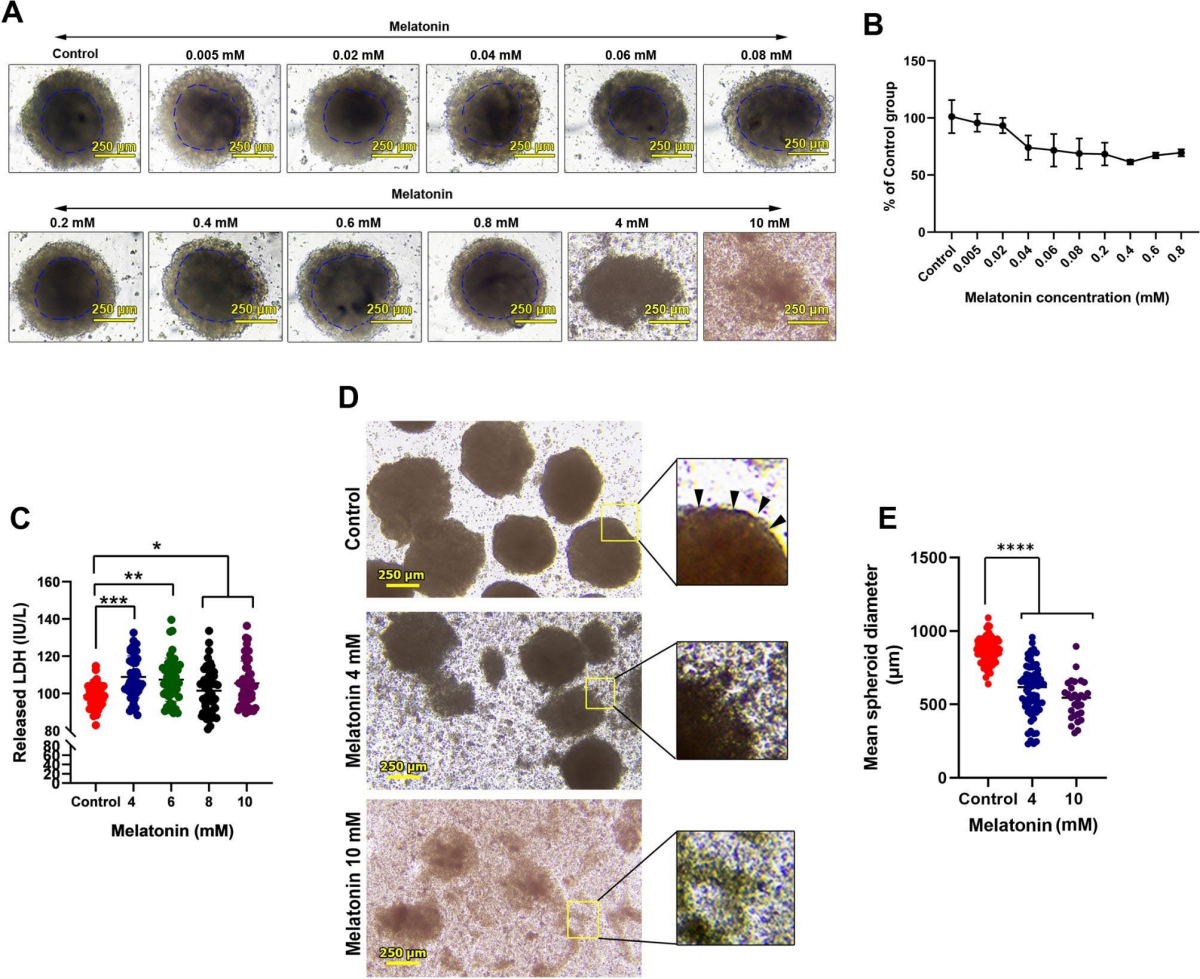
Bright-field images of 3D colon cancer tumoroids after being exposed to different concentrations of melatonin (**A**). An inner compact zone is evident at the center of each tumoroids. 48-hour treatment with melatonin did not affect the integrity of spheroids. Measuring survival rate using an MTT assay (**B**). The survival rate was reduced by increasing the melatonin concentration from 0.005 to 0.8 mM (n = 12). As shown in panel A, melatonin at this range did not affect the integrity of tumoroids. Measuring survival rate using an LDH leakage assay (**C;** n = 12). Tumoriods were treated with higher melatonin concentrations, 4 to 10 mM, for 48 h. Data confirmed the significant increase of supernatant LDH compared to the control tumoroids, indicating reduced cell membrane integrity in the presence of melatonin. Bright-field images of tumoroids 48 h after being treated with melatonin (**D**). In non-treated tumoroids, dense and compact tumoroids structures with flattened cells (arrowheads) at the periphery zone are evident. In melatonin-treated groups, cells within the tumoroids parenchyma are disaggregated and these features were more evident in group melatonin 10 mM. Calculation of mean tumoroids diameter (**E;** n = 12). Melatonin can significantly reduce the diameter of 3D colon cancer tumoroids after 48 h in in vitro conditions. One-Way ANOVA with Tukey post-hoc analysis. *p < 0.05; **p < 0.01; ***p < 0.001; and ****p < 0.0001

In this study, an in vitro 3D tumoroid system was used to evaluate the possible tumoricidal effects of melatonin (Fig. 1A-E). Bright-field imaging revealed a typical tumoroid structure in the control group with an inner compact core after 48 h. The tumoroid mass was wrapped by flattened cells at the periphery. Incubation with lower doses of melatonin, ranging from 0.005 to 0.8 mM did not affect the integrity of CRC tumoroids after 48 h (Fig. 1A). Tumoroids exposed to melatonin at higher doses (4 and 10 mM) exhibited loosely appearance especially in group 10 mM melatonin (Fig. 1A) These data demonstrate that lower doses of melatonin cannot affect the integrity of 3D cancer cell structure in in vitro conditions.

An MTT assay was used to assess the survival rate in melatonin-treated tumoroids after 48 h (Fig. 1B). Data indicated that the metabolic activity (viability) of tumoroid cells was reduced by increasing melatonin concentration from 0.005 to 0.8 mM in which the survival rate was slightly reduced compared to the non-treated control tumoroids. Despite the reduction in survival rate, the integrity of CRC tumoroids was intact. Thus, these data indicate that CRC tumoroid integrity was not affected in the groups that received fewer melatonin contents despite the slight reduction of tumoroid cell viability. Based on these data, the oncostatic/tumoricidal properties of melatonin were also examined in the presence of higher doses of melatonin, ranging from 4 to 10 mM, using an LDH release assay (Fig. 1C). Data indicated statistically significant differences in the levels of LDH in melatonin-treated groups when compared to the control group (*p*_Control VS. 4 mM<0.001;_*p*_Control VS. 6 mM<0.01;_*p*_Control VS. 8 mM<0.01;_ and *p*_Control VS. 10 mM<0.05_). These data demonstrate that the levels of released LDH and necrotic changes were increased in the CRC tumoroids after being incubated with higher doses of melatonin.

To investigate whether higher doses of melatonin can disintegrate the structure of tumoroids, melatonin-treated CRC tumoroids were visualized under light microscopy in 4- and 10-mM melatonin groups (Fig. 1D). According to the data, higher doses of melatonin can reduce the integrity of tumoroids, especially in the groups that were exposed to 10 mM melatonin. In the control tumoroids, apparent compact and dense structures were evident with less detached cells in the periphery (Fig. 1D; black arrowheads). While in 4- and 10-mM melatonin groups, the number of detached cells was increased (Fig. 1D). Along with these features, tumoroids lost their integrity and flattened the outermost cell layer in which isolated micro-sized cell aggregates can be easily detected on the plastic surface. These features were more evident in the group exposed to 10 mM melatonin.

### Tumoroid diameter was reduced by increasing doses of melatonin

To this end, the mean diameter size of tumoroids was measured after treatment with melatonin compared to the control group (Fig. 1E). Data showed that the mean size of CRC tumoroids was significantly reduced after exposure to melatonin at doses 4 and 10 mM (*p*_Control VS. 4 and 10 mM<0.0001_). It was suggested that melatonin can reduce the survival rate of cells within the tumoroids in both lower and higher doses, resulting in the reduction of CRC tumoroid expansion. However, the tumoroid integrity was not affected in groups exposed to lower doses of melatonin. In contrast, melatonin can increase cell membrane permeability and disrupt tumoroid integrity at higher doses like 4 and 10 mM.

### Melatonin reduced angiogenic activity in CRC tumoroids

**Fig. 2:**
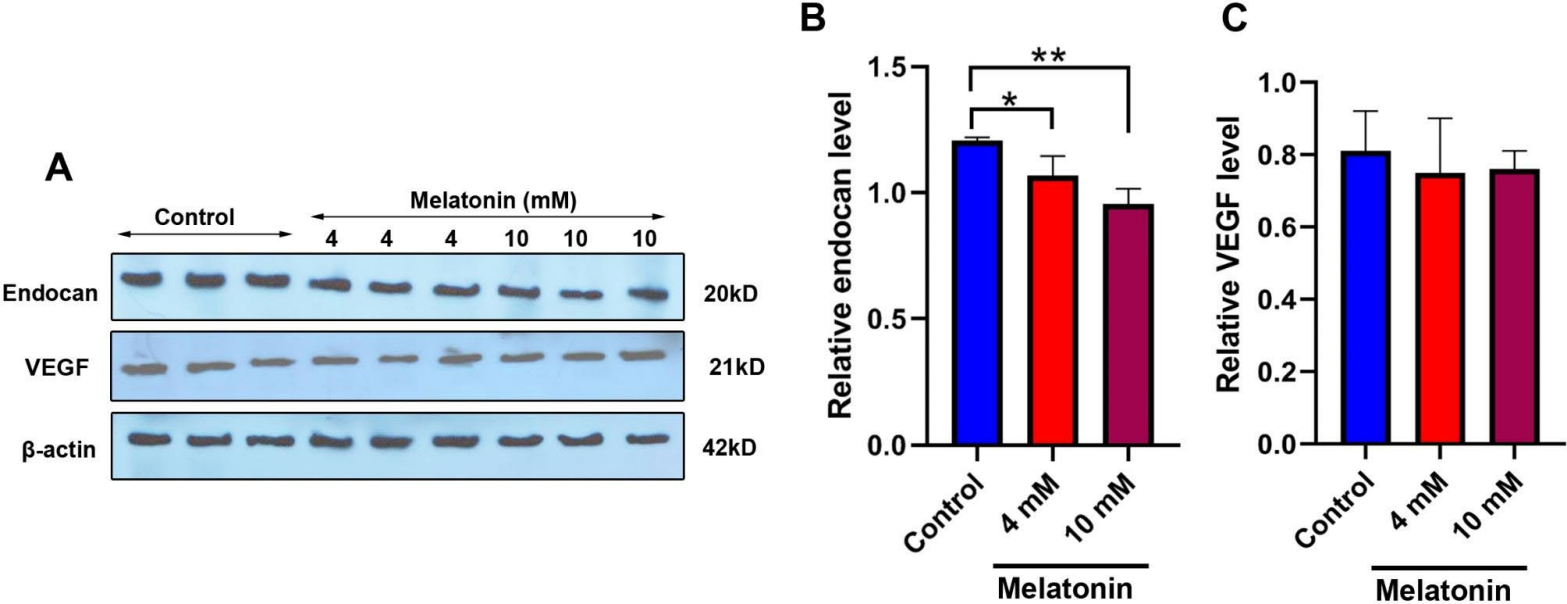
Monitoring protein levels of endocan and VEGF using the western blot technique (**A-B**). Treatment with melatonin with doses of 4 and 10 mM significantly reduced endocan levels within the tumoroids after 48 h compared to the non-treated tumoroids. Based on the data, melatonin did not alter protein levels of VEGF in 4 and 10 mM melatonin groups compared to the control tumoriods (n = 3). One-Way ANOVA with Tukey post-hoc analysis. *p < 0.05 and **p < 0.01

The levels of endocan and VEGF were measured using western blotting to monitor the angiogenesis status in melatonin-treated CRC tumoroids (Fig. 2A-B). According to the data, 48-hour incubation of tumoroids with melatonin decreased protein levels of endocan compared to the control group (p < 0.05). Statistical analyses revealed a lack of significant differences in endocan levels between the groups that received 4- and 10-mM melatonin (p > 0.05). In contrast to endocan, non-significant differences were obtained in terms of VEGF levels in 4 and 10 mM melatonin compared to the control group (p > 0.05). These data demonstrated that protein levels of certain angiogenic factors such as endocan are reduced in tumoroid structure after exposure to higher doses of melatonin. Taken together, the suppression of certain angiogenesis factors would be at least one of the possible mechanisms in which melatonin can inhibit the expansion of the tumoroid system.

### Melatonin can loosen colon tumoroid integrity

**Fig. 3:**
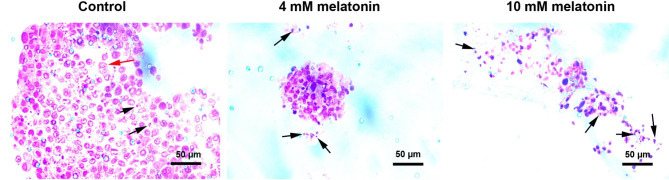
Hematoxylin-Eosin staining. In control tumoroids, dense parenchyma is evident with dividing (red arrow) and necrotic cells (black arrows). In melatonin-treated tumoroids, the number of necrotic cells increased. In groups treated with 4 mM melatonin, small-sized cell aggregates with prominent necrotic changes can be detected. Based on the data, 10 mM melatonin efficiently disaggregated cells within the tumoroids parenchyma with numerous necrotic changes

In this study, H & E staining was performed to monitor CRC tumoroid parenchyma after being exposed to 4- and 10-mM melatonin. According to bright-field images, the development of CRC tumoroids using HT-29, HUVECs, and HFFF2 cells produced a dense parenchyma with tightly juxtaposed cells (Fig. 3). Data indicated the existence of numerous dividing cells inside the CRC tumoroids, indicating a high-rate mitotic index (Fig. 3; red arrows). In the inner layer, necrotic cells can be also detected (Fig. 3; black arrows). Treatment of CRC tumoroids with melatonin led to the loss of tumoroid integrity and cell-to-cell connection in which separate small-sized cell aggregates with an increasing number of necrotic cells are visible in the melatonin 4 mM group (black arrows). These values were more evident in the group that received 10 mM melatonin. In the group treated with melatonin 10 mM, a loose matrix with numerous necrotic cells is evident. These data showed that melatonin can increase the number of necrotic cells within the CRC tumoroids in a dose-dependent manner.

## Discussion

The primary aim of this study was to assess the tumoricidal properties of melatonin on 3D CRC tumoroids in in vitro conditions. To mimic the complexity and heterogeneity of colorectal cancers, three cell lines, including HT-29, HUVECs, and HFFF2 cells, were used. Using the current protocol, we successfully developed a 3D culture system to monitor the anti-cancer properties of melatonin. Bright-field analysis exhibited an inner compact zone at the center of CRC tumoroids that is surrounded by the outermost cellular layer. Treatment with melatonin at the range between 0.005 to 0.8 mM did not affect the tumoroid integrity while higher doses, 4- and 10-mM, led to the loss of tumoroid integrity and reduction of diameter size. These features coincided with the reduced cell density and promotion of necrotic changes within the parenchyma. Proteomic analysis indicated an inhibitory effect of melatonin on the angiogenic behavior of cancer cells via the reduction of endocan in a dose-dependent manner. However, melatonin did not alter protein levels of VEGF in CRC tumoroid system.

Angiogenesis or vascularization plays a crucial role in tumor mass expansion and metastasis rate [33]. In this regard, numerous studies have been conducted in terms of the regulation of the angiogenesis signaling pathway [34]. It has been shown that tumoroids generally possess three district concentric zones within their structures. The external surface includes highly proliferating cells with metastatic behavior while in the middle and central zones, quiescent cancer cells and necrotic cells can be detected, respectively [35]. Due to the limited diffusion of oxygen and nutrients into the tumoroid inner zones, cells acquired a quiescence state to adapt to the environmental conditions. In 3D tumoroid structures with an average diameter of 500 µm or more, the existence of hypoxia in the innermost zone led to the promotion of angiogenesis factors HIF-1α, P-glycoprotein, and VEGF, leading to cancer cell resistance and recapitulating in vivo conditions [35, 36]. Endocan is another pro-angiogenesis factor that is produced by ECs in different tissue types. It was suggested that the over-expression of endocan is associated with the expansion and metastasis of cancer cells [35]. Here, we found that the levels of endocan were higher in control tumoroids in comparison with the melatonin-treated groups. One possible mechanism would be that the inner dark necrotic area contains higher levels of pro-inflammatory cytokines such as tumor necrosis factor-α and interleukin-1, leading to the induction of endocan and VEGF-A [37, 38]. It has been indicated that the exposure of hypoxic cells to melatonin can reduce the production of angiogenesis factors [39]. Melatonin can diminish the expression of HIF-1α via the neutralization of reactive oxygen species and regulation of Sphingosine kinase 1 activity and TGF-β signaling pathway [40, 39, 41]. The reduction of reactive oxygen species and down-regulation of VEGF were documented in hypoxic ECs treated with melatonin [42]. The inhibition of STAT3 by melatonin can also diminish the production of erythropoietin, reactive nitrogen species, and VEGF [42].

To the best of our knowledge, numerous studies explored the effect of melatonin on angiogenesis status in a 2D cell culture setting rather than 3D tumoroid systems. In an experiment conducted by Zhang and co-workers, 48-hour incubation of human gastric adenocarcinoma SGC7901 cells with 0.0001 mM melatonin for 48 hours led to an increase in endocan levels related to non-treated cells [43]. They claimed simultaneous reduction of intracellular alkaline phosphatase and lactate dehydrogenase and inhibition of the dedifferentiation phenomenon and resistance in SGC7901 cells. In the most of previously conducted using 2D colon cell culture experiments, melatonin was used in lower concentrations compared to the current study using 3D colon cancer tumoroids [44, 45]. Unlike the 2D culture system, it seems that this strategy is arguable in colon cancer tumoroids as it can increase the possibility of tumor mass expansion and cancer cell metastasis.

In contrast to previous studies, higher doses of melatonin (4–10 mM) were also applied in the present experiment. Although almost all previous experiments have confirmed the direct oncostatic properties of melatonin on tumor cells cultured in a 2D culture system, it seems that similar melatonin concentrations are not effective to exert anticancer properties on cancer cells within the tumoroid structure. As shown in bright-field images, the integrity of tumoroids exposed to different ranges of melatonin from 0.005 to 0.8 mM was not affected, indicating the lack of an oncostatic effect. However, treatment with higher doses of melatonin (4–10 mM) led to the disaggregation of tumoroids into a single-cell suspension. Due to the development of parenchyma in various solid tumors, it seems that present data reflect appropriately the in vivo-like efficiency of melatonin compared to the 2D culture system. It has been shown that melatonin can reduce the production of extracellular matrix components (ECM), especially type I collagen and fibronectin in fibroblasts via the inhibition of TGF-β1 signaling pathways such as SMADs and Akt, ERK1/2, and p38 [46]. ECM can strengthen the tumor parenchyma via the interaction between cell-surface receptors and several motifs within the ECM structure. These features per se help the cells to maintain cell-to-cell integrity [47]. Current data indicated enhanced necrotic changes in the structure of colon tumoroids indicated with fragmented nuclear parts. Previously, the inhibitory effects of melatonin were proved on cell proliferation and dynamic growth via arresting cells at the G0/G1 state and suppression of autophagy response via down-regulation of AMPKα1 expression [48].

The current study faces several limitations and it is suggested that future experiments should address these issues for a better understanding of the oncostatic effects of melatonin on 3D colon tumoroids. Here, we just monitored protein levels of endocan and VEGF and it would be better for future studies to measure the expression and protein levels of other factors related to the angiogenesis behavior of cancer cells within the CRC tumoroids. By using specific staining, the exact location of each cell type within the tumoroids pre- and post-melatonin treatment can be addressed.

## Conclusion

3D tumoroid system is an efficient modality for the evaluation of anti-cancer compounds compared to the conventional 2D culture system. Here, we evaluated the oncostatic properties of melatonin in close-to-real colon cancer tumoroids containing three different cell lines including adenocarcinoma HT29 cells, fetal foreskin HFFF2 fibroblasts, and HUVECs to mimic in vivo-like conditions. Data indicated that melatonin can efficiently reduce the integrity of organotypic tumor mass via the suppression of certain angiogenesis factors such as endocan with simultaneous induction of necrotic changes. These features confirmed the high-throughput tumoricidal properties of melatonin in complex colon tumor niches.

## Data Availability

The datasets used and/or analyzed during the current study are available from the corresponding author upon reasonable request.

## Abbreviations

2D: Two-dimensional
3D: Three dimensional
BFGF: Basic fibroblast growth factor
CRC: Colorectal cancer
DMSO: Dimethyl sulfoxide
ECM: Extracellular matrix
FBS: Fetal bovine serum
H&E: Hematoxylin-Eosin
HFFF2: Human foreskin fibroblasts
HRP: Horseradish peroxidase
HUVEC: Human umbilical vein endothelial cells
LDH: Lactate dehydrogenase
PBS: Phosphate buffered saline
PBST: Phosphate-buffered saline with Tween 20
PVDF: Polyvinylidene fluoride
VEGF: Vascular endothelial growth factor
